# Lorlatinib and Amivantamab: A Paradigm Shift in *EGFR* and *ALK* Positive NSCLC, with More Effective but More Toxic Treatments Requiring a Well-Structured Shared Decision Making

**DOI:** 10.32604/or.2026.072992

**Published:** 2026-03-23

**Authors:** Paolo Maione, Valentina Palma, Cesare Gridelli

**Affiliations:** 1 Division of Medical Oncology, S.G. Moscati Hospital, Avellino, 83100, Italy; 2 Division of Medical Oncology, Università degli Studi della Campania “Luigi Vanvitelli”, Napoli, 80121, Italy

**Keywords:** Non-small cell lung cancer, shared-decision making, amivantamab, lorlatinib, osimertinib, alectinib, brigatinib

## Abstract

After about 20 years of exciting improvements in treatment efficacy outcomes of advanced epidermal growth factor receptor (*EGFR*) mutant and anaplastic lymphoma kinase (*ALK*) rearranged non-small cell lung cancer (NSCLC), also combined with a progressively better safety profile, from chemotherapy to new generation tyrosine kinase inhibitors (TKIs) (osimertinib, alectinib, brigatinib), the recent MARIPOSA and CROWN trials have changed this trend. For the first time in the history of *EGFR* and *ALK* treatments, we must face the issue of being a step behind in terms of toxicity profile. The combination of amivantamab plus lazertinib in *EGFR* mutant NSCLC, and lorlatinib in *ALK* rearranged NSCLC, has improved efficacy outcomes as never before. The story would be easy and totally positive if these two innovative, amazing treatments were not associated with new peculiar features in safety profiles that must be discussed with patients, because they potentially affect their quality of life. When treating these patient populations, the peculiar safety profiles of amivantamab plu lazertinib and lorlatinib require a well-structured shared decision making, “where and when”, both the high probability of a longer survival and the risk of worse quality of life must be well announced and explained to our patients before the shared final treatment choice.

## 1 Introduction

About twenty years ago, the history of advanced non-small cell lung cancer (NSCLC) treatment changed forever. An oral epidermal growth factor receptor (*EGFR*)—tyrosine kinase inhibitor (TKI), gefitinib, was demonstrated to achieve brilliant clinical and radiological response (sometimes “Lazarus responses”) in patients whose NSCLCs harboured activating mutations in the EGFR gene, and buried chemotherapy in this patient population [1,2]. Something never seen before in the treatment of this advanced disease was reported, characterized by deep tumor response, survival prolongation, and excellent quality of life. For the first time, lung cancer oncologists observed something in their patients that we may define as “clinical benefit”, i.e., efficacy, symptom improvement with a mild toxicity profile in the majority of cases, and oral therapy. The oncogene addicted disease had been discovered in lung cancer treatment. This exciting treatment experience opened the road towards the search for other oncogene addicted diseases, and nowadays we have a relevant proportion of advanced NSCLC patients, mainly never smokers, who are affected by oncogene addicted disease. In fact, *EGFR*, anaplastic lymphoma kinase gene (*ALK*), reactive oxygen species (ROS) proto-oncogene 1 receptor tyrosine kinase (*ROS*-1), rearranged during transfection gene (*RET*), proto-oncogene B-Raf serine/threonine kinase (*BRAF)*, neurotrophic tyrosine receptor kinase (*NTRK*), mesenchymal epithelial transition (*MET*) proto-oncogene receptor tyrosine kinase exon 14 skipping mutations, human epidermal growth factor receptor 2 (HER2) gene mutations, kirsten rat sarcoma (KRAS) gene mutations and neuregulin-1 gene (*NRG1*) fusions diseases, are characterized by excellent treatment outcomes and really surprisingly changed natural history [3]. Oncogene addicted NSCLC patients can be treated with targeted agents, with excellent activity and efficacy outcomes, and with a generally excellent quality of life. A great proportion of these patients can live their lives almost as if they were not affected by cancer, having their oral therapy at home and with very few visits to the hospital for long periods of time. For *EGFR* TKIs, the search for better treatment options moved from first-generation TKIs, gefitinib and erlotinib [4], to second-generation irreversible TKIs, such as afatinib [5], and finally to third-generation irreversible and selective TKIs, such as osimertinib [6,7]. The other “big oncogene addicted disease” to be discovered was *ALK*-rearranged NSCLC [8], with the first-generation *ALK* inhibitor crizotinib clearly having improved chemotherapy outcomes in this clinical context with excellent efficacy, safety, and quality of life results [9]. Outcomes of crizotinib have been further improved with clinical development of the second-generation of *ALK* inhibitors, alectinib [10,11] and brigatinib [12,13], that have achieved really relevant efficacy and quality of life outcomes to be considered the best (in terms of progression-free survival) in the panorama of oncogene addicted NSCLC, even better than in *EGFR* mutant NSCLC. However, all patients with *EGFR* mutant and *ALK* rearranged advanced NSCLC finally experience drug-resistance, clinical and radiological progression, and finally death. Thus, the research has focused on new forms of treatment. Better progression-free-survival (PFS) and overall survival (OS) outcomes have been achieved, in a phase III randomized trial named MARIPOSA, by combining an oral third-generation TKI (lazertinib) with the bi-specific *EGFR-MET* monoclonal antibody amivantamab, for *EGFR* mutant NSCLC [14,15]. In *ALK* rearranged disease, the third-generation *ALK* inhibitor lorlatinib has achieved in a phase III randomized trial named CROWN, compared with crizotinib, PFS outcomes never seen before, not only in this clinical context but also in the entire universe of targeted therapies for solid tumors [16,17]. However, these two extraordinary efficacy outcomes are not considered a success because they are associated with a step back in terms of safety profile and quality of life. In other words, they may be considered as “bitter victories”, with efficacy outcomes that cannot be ignored and that must be proposed to our patients, but with the other side of the coin, a worse toxicity profile, that must be equally considered.

## 2 Amivantamab Plus Lazertinib vs. Osimertinib in Advanced *EGFR* Mutant NSCLC Treatment

Amivantamab is an *EGFR-MET* bispecific antibody with multiple mechanisms of action. In fact, it exerts its action by ligand blocking, receptor degradation, and immune actions by recruitment of effector cells, such as natural killer cells [18,19]. Amivantamab binds to *EGFR* extracellularly, bypassing intracellular mutations (including those at the TKI catalytic domain), and moreover, its action against *MET* activity aims to react against mechanisms of resistance to *EGFR*-TKIs. Its activity against *MET* is particularly relevant; in fact, *MET* amplification is a dominant *EGFR*-independent mechanism of resistance, accounting for 15%–22% of resistance to first and second-generation *EGFR*-TKIs and 5%–15% to third-generation agents like osimertinib [20]. Lazertinib, as osimertinib, is a selective, oral third-generation *EGFR*-TKI, effective in *EGFR* mutant NSCLC both with activating and T790M resistance mutations [21]. Thus, it is intuitive that combining amivantamab and lazertinib means to target both the extracellular and intracellular catalytic *EGFR* domains, and this mechanism has already been demonstrated to produce a synergistic clinical benefit [22]. Consequently, the authors of the phase III randomized trial named MARIPOSA [14] mainly based their scientific rationale to test the combination of amivantamab plus Lazertinib, against monotherapy with osimertinib, as first-line treatment of advanced *EGFR* mutant NSCLC, on the attempt to proactively target mechanisms of resistance to osimertinib. The combination of amivantamab plus lazertinib achieved a significantly longer median PFS compared with osimertinib alone (primary endpoint of MARIPOSA trial; 23.7 vs. 16.6 months, respectively, with a hazard ratio (HR) of 0.70 (95% CI 0.58–0.85; *p* < 0.001). Moreover, the combination has met the final pre-specified secondary endpoint of OS, demonstrating clinically meaningful and statistically significant improvement in OS vs. osimertinib [15]. At a median follow-up of 37.8 months, median survival resulted in 36.7 months for osimertinib and not reached for the combination, with a hazard ratio for death of 0.75 (95% CI, 0.61–0.92; *p* < 0.005). Moreover, the survival rate at three years was 60% in the amivantamab plus lazertinib group compared with 51% in the osimertinib arm. However, the combination of amivantamab and lazertinib resulted in more effective outcomes in terms of PFS and OS, as more toxic. The combination resulted in a worse safety profile in terms of rate of grade 3 or higher adverse events, mainly paronychia and skin rash (75% vs. 43%, for amivantamab plus lazertinib and osimertinib alone, respectively). The topic of infusion-related reactions and venous thromboembolic adverse events typically associated with amivantamab administration was confirmed in the MARIPOSA trial, occurring in 63% and 37% of the patients treated with amivantamab–lazertinib and osimertinib, respectively.

However, it is important to underline that the majority of infusion reactions occurred during the first infusion and the majority of thromboembolic events during the first 4 months, suggesting that a proactive approach in clinical practice may reduce these adverse events in the future (i.e., increased use of prophylactic steroids at first infusions and prophylactic anticoagulation). Specifically, a very recent phase II trial named SKIPirr showed that prophylactic treatment with dexamethasone 8 mg oral BID during the two days preceding cycle 1 day 1 and 1 h prior to infusion of amivantamab on cycle 1 day 1 resulted in an about 3-fold reduction in infusion-related reactions (IRRs) compared with standard management (from 67.4% to 22.5%). Moreover, in the SKIPirr trial, IRR rate reduction occurred for all grades of IRRs [23]. With the aim of preventing dermatologic adverse events during amivantamab treatment, the phase 2 COCOON study is evaluating enhanced preventive dermatologic management in patients treated with first-line amivantamab and lazertinib. The COCOON dermatologic management regimen consists of oral doxycycline or minocycline for 12 weeks, followed by topical clindamycin lotion on the scalp, chlorhexidine on the nails, and a ceramide-based moisturizer on the body and face. The first interim analysis showed that during the first 12 weeks of amivantamab plus lazertinib treatment, the incidence of grade 2 or higher dermatologic adverse events was 38.6% in patients who were randomized to receive the COCOON prophylactic regimen compared with 76.5% in patients who received standard-of-care dermatologic management (OR, 0.19; 95% CI: 0.09–0.40; *p* < 0.0001) [24]. It is to underline that, in the PALOMA-3 phase III randomized trial, subcutaneous amivantamab plus lazertinib recently demonstrated non-inferiority to intravenous amivantamab plus lazertinib, with a significant reduction in the rate of infusion-related reactions (13% vs. 66%) compared with intravenous amivantamab [25]. As a consequence, subcutaneous amivantamab will early become the preferred way of administering this drug with a significant mitigation of its safety profile. In the near future of our clinical practice, we can also guess that the reduction of the IRR rate available with the COCOON prophylaxis may be enhanced by the adoption of the subcutaneous route of administration of amivantamab. In fact, as suggested by Saw and Bironzo prophylaxis should not be omitted when using the subcutaneous route of administration of amivantamab, for a maximal improvement of the safety profile [26]. On the contrary, we may argue that in clinical practicethe prophylactic regimens suggested for amivantamab plus lazertinib will not be so easy to apply, and mainly out of pocket for patients. In conclusion, the combination of amivantamab plus lazertinib produced a significant and clinically relevant PFS and OS improvement over osimertinib alone, with an absolute 3-year benefit in the probability of being alive of 9%. This relevant efficacy outcome is at the cost of significant symptomatic, early and long-lasting toxicity, requiring intensive medical prevention and management, and impacting the daily quality of life of patients during a period when the disease is generally well-controlled.

## 3 Lorlatinib Instead of Alectinib or Brigatinib in Advanced *ALK* Rearranged NSCLC Treatment

Lorlatinib, a third-generation *ALK* inhibitor, was compared with crizotinib, as first-line treatment, in the phase III CROWN study [16,17]. The percentage of patients who were alive without disease progression at 12 months was 78% (95% CI, 70 to 84) in the lorlatinib group and 39% (95% CI, 30 to 48) in the crizotinib group (HR for disease progression or death, 0.28; 95% CI, 0.19 to 0.41; *p* < 0.001). The response rate resulted 76% (95% CI, 68 to 83) for patients treated with lorlatinib and 58% (95% CI, 49 to 66) for those treated with crizotinib; among those with measurable brain metastases, 82% (95% CI, 57 to 96) and 23% (95% CI, 5 to 54), respectively, had an intracranial response, and 71% of the patients who received lorlatinib had an intracranial complete response [16]. With a median duration of follow-up for PFS of 60.2 months for patients treated with lorlatinib and 55.1 months for patients treated with crizotinib, the HR for disease progression or death with lorlatinib vs. crizotinib resulted in 0.19 (95% CI, 0.13 to 0.27). Median PFS was not reached with lorlatinib and 9.1 months with crizotinib. The 5-year PFS was 63% for patients treated with lorlatinib and 8% for those treated with crizotinib [17]. Among patients with brain metastases at trial enrollment, the benefit in favour of lorlatinib was even greater, resulting in an HR for disease progression or death with lorlatinib vs. crizotinib of 0.08 (95% CI, 0.04 to 0.19), and the median PFS was not reached for patients treated with lorlatinib and 6.0 months for those treated with crizotinib. Thus, the CROWN study demonstrated an impressive and never-before-observed magnitude advantage in PFS, the primary objective of the trial, of lorlatinib vs. crizotinib, indirectly better than that reached by second-generation inhibitors brigatinib and alectinib. The PFS benefit reported for lorlatinib in the CROWN study, exceeding 5 years, is also the longest reported PFS in advanced NSCLC to date.

Lorlatinib is characterized by low P-1-glycoprotein-mediated efflux capacity and good brain penetration. P-1-glycoprotein (P-1-gp) is a major efflux transporter of the blood-brain barrier that limits intracranial exposure and CNS activity of some drugs. It exports substrates back towards the blood, limiting their capacity to accumulate in the brain. Lorlatinib is a weak P-1-gp substrate; therefore, it can remain within the CNS and does not get exported back into the blood [27]. It achieves meaningful concentrations in the brain, thereby providing strong antitumor efficacy. But the increased brain pharmacological accumulation is also related to adverse events at the CNS level. In fact, the studies show that cognitive, mood, psychotic, and language effects are the most common adverse events of lorlatinib on the central nervous system. Moreover, as observed by Dagogo-Jack et al., in the real-world practice, lorlatinib-related neuro-cognitive adverse events incidence may be influenced and increased (compared to clinical trials) by multiple factors, including brain metastases, brain radiation, psychiatric illnesses, and use of neurotropic medications [28]. Jia and Jen also discussed this topic, underlining that for patients with the above-mentioned risk factors for neurocognitive adverse events, second-generation *ALK inhibitors* (i.e., alectinib and brigatinib) should be the best treatment choice instead of lorlatinib [29]. Moreover, weight gain and dyslipidemia [30] are frequent and often also severe adverse events that negatively influence the quality of life of the patients. Lorlatinib has a unique side effect profile compared with other *ALK* TKIs. Symptomatic side effects like limb swelling (edema), weight gain, numbness or tingling (neuropathy), and mild changes in mood, speech, thinking, or behavior can frequently occur, and often can be managed with dose adjustment [31,32]. In particular, increased body weight occurred in 44% of the patients treated with lorlatinib in the CROWN study, with about half of them reporting severe weight gain, defined as an at least 20% increase in body weight over baseline [16,17]. It is also to remind that anxiety and depression occurred in 21% of patients (1% severe) and that hallucinations (psychotic effects), occurred in 5% of patients. Thus, potential severe neuro-psychiatric and metabolic adverse events are to be considered the Achilles’ heel of lorlatinib treatment, and, in our opinion, have to be clearly discussed before treatment starts for a shared treatment choice. On the contrary, the safety profile of second-generation *ALK* inhibitors alectinib and brigatinib is particularly favourable, with the most common adverse events being laboratory abnormalities (more frequent with brigatinib). Among non-laboratory adverse events, are to mention hypertension (occurring more frequently with brigatinib [12,13]) and peripheral edema (occurring more frequently with alectinib [10,11]).

## 4 Are There Any Clinical or Molecular Factors to Select Patients for More Effective but More Toxic Treatments in *EGFR* Mutant and *ALK* Rearranged Diseases?

Several clinical and molecular factors have been searched for to escalate or de-escalate the intensity of treatment for EGFR mutant NSCLC [33]. However, current data show only a prognostic and not a predictive role for some of them [34]. The presence vs. absence of liver and/or brain metastases, presence vs. absence of TP53 co-mutation, positive vs. negative circulating tumor DNA, seem to show subgroups of patients with the worst prognosis independently of the treatment, and the greater benefit with the more toxic combination is observed also in the subgroups of patients with better prognosis. Thus, when communicating with our patients, subgroup analyses can be used to better clarify the prognosis, and the absence of negative prognostic factors may indicate to patients that the outcome in terms of PFS may also be impressively long with osimertinib monotherapy. For *ALK* rearranged NSCLC, the feeling that good-prognosis patients may benefit from very long PFS and OS times from brigatinib [12,13] or alectinib [10,11], as with lorlatinib, also exists. However, the lack of direct comparisons among second- vs. third-generation *ALK*-TKIs does not give us the possibility to scientifically affirm this hypothesis. In other words, lorlatinib may be superior in terms of efficacy in both bad and good prognosis subgroups of patients. However, also in younger and fit patients, especially when dealing with *ALK-positive* NSCLC, some prognostic factors (such as rearrangement variants, sites of disease, co-mutations) should be used to discuss upfront TKI choice. Both for *EGFR* mutant and *ALK* rearranged NSCLC, in our opinion, a common sense approach, specific for elderly patients, probably if aged more than 75, but certainly if aged more than 80 years, should be used. In other words, osimertinib and alectinib or brigatinib are to be considered excellent treatments for advanced NSCLC of elderly and very elderly patients (high efficacy, and safety profile particularly suitable to that age) [35], and an attempt to increase efficacy at the cost of an increase in toxicity in such a special population, very often characterized by polypharmacotherapy, frailty and lack of social and familiar support, may result in a frequent destruction of the elderly person’s dignity. In our opinion, amivantamab plus lazertinib and lorlatinib should be used in a selected minority of elderly patients aged more than 75 years, and almost always in those aged more than 80 years.

## 5 Shared Decision Making as One of the Main Selection Drivers in *EGFR* Mutant and *ALK* Rearranged NSCLC Treatment: A Structured Form Is Needed

In conclusion, clinical and molecular prognostic factors, useful to choose among more toxic and efficacious vs. less toxic and efficacious treatments in *EGFR* mutant and *ALK* rearranged NSCLC, are, however, not enough. Thus, individual patient opinion, within a shared decision making (SDM), becomes a crucial approach to choose the best treatment for every single patient [36,37]. Structured and validated tools for SDM do not yet exist for this clinical context [38,39]; thus, in our opinion, it is time to improve on this issue. Here, we propose a form of SDM for patients affected by *EGFR* mutant or *ALK-rearranged* advanced NSCLC. For an optimal SDM, before speaking about candidate treatments, the physician should briefly interview the patient on her/his social, family, economic, and working features. Then, the first element to clarify and explain is the efficacy of the available therapeutic options, in terms of the difference in median overall survival or survival rate (at 3 or 5 years). If available, the probability of being alive at 5 years is an outcome better understood by patients, and also the preferred outcome to achieve (the probability of being a long survivor). However, the median survival can be communicated to patients to give them an idea of the most probable duration of their survival. The second element is the description of the safety profile of therapeutic options (particularly symptomatic toxicities and visible adverse events like skin toxicity), route of administration (oral vs. subcutaneous vs. intravenous), number of hospital visits per month and per year. In the case of amivantamab plus lazertinib, skin toxicity must be well announced as the risk of thrombovenous embolism and of infusion-related reactions (however, specifying their reduced rate with the subcutaneous route of administration), with the following needed polypharmacotherapy for the prophylaxis of these adverse events. For lorlatinib, the higher risk of neurocognitive, psychiatric, and metabolic adverse events (also weight gain) must be clearly announced to patients. After efficacy and toxicity information, the third phase is a fusion of the two previous phases, with the exploration of the psychological approach towards the extent of the prolongation of survival that the patient would intend to trade off with the risk of a certain type of toxicity. After this informative phase, the final decision should not be taken at the first visit, except when the patient requires immediate treatment and has already decided. A second visit should be scheduled within 5 days (from 3 to 5 days) for the final decision, to let the patient to metabolize the given information. This time interval, in our opinion, is sufficient for the patient to metabolize the given information, to listen to her/his own intimate feelings, and if necessary and desired, to collect further opinions and data on the topic from other sources, but not excessively long to delay cancer treatment.

At the end of the first visit, with the patient already out of the visit room, the physician should make and write down his treatment choice on the basis of the information given by the patient on “her/his world”, of the reactions and feedbacks of the patient to efficacy and toxicity data of the treatment options, obviously in light of his scientific knowledge and practical experience. The treatment choice should be recorded and not communicated, but used at the second visit.

At the second visit, two scenarios may be found. The first, easier, with the patient having already and firmly decided, with the SDM rapidly concluded. The second patient, who arrives confused. In this case, the recorded physician’s treatment choice should be the driver, with the patient gently guided and suggested to follow that choice (which, however, derives from the physician’s scientific knowledge and experience but also from the previous getting acquainted with the patient) (Table 1).

**Table 1:** Structured SDM among two treatment options (one more efficacious but also more toxic than the other)

**Phase**	**Goal**
Visit 1	
Patient’s world	Brief description of patient’s life (familiar, socio-economic and working features and hobbies)
Treatment options: Efficacy	Description of efficacy data in terms of PFS and, if available OS (survival rate or median survival)
Treatment options: Toxicity	Description of toxicity data, mainly symptomatic and visible toxicities
Trade off efficacy/toxicity	Understand the psychological approach of the patient in terms of survival prolongation and quality of life
Visit 1 ended	
Physician’s choice for that patient (immediately after visit 1)	Physician, on the basis of patients’ description of “her/his world”, of reactions to efficacy and toxicity data, and obviously of his clinical experience, makes a treatment choice, recorded but not communicated to the patient
Visit 2, after 3–5 days	
Patient decision	
Patient gives his decision	SDM concluded successfully
Patient confused	Patient conducted towards the physician’s choice with further explanation. SDM concluded successfully

Note: PFS: progression-free survival; OS: overall survival; SDM: shared-decision making.

## 6 Conclusion

Although the results of MARIPOSA and CROWN trials are of extreme relevance, in our opinion, the adoption of these treatments (amivantamab plus lazertinib and lorlatinib) indiscriminately to the majority of patients is a wrong approach that may negatively change the oncogene addicted NSCLC clinical context as we have been used to experience it as physicians and patients in the last twenty years. A clinical context characterized not only by efficacy but also by excellent quality of life. More toxic treatments should be introduced cautiously and with SDM whenever possible. Very recently, the combination of platinum-based chemotherapy plus osimertinib has also been demonstrated to produce better outcomes in terms of overall survival compared to osimertinib alone (FLAURA 2 trial) in the first-line treatment of *EGFR* mutant advanced NSCLC [40]. The safety profile of this combination is more favourable compared with amivantamab plus lazertinib, but worse than osimertinib alone. Collocating this treatment option is perhaps a mid-way choice, but requires a wide discussion and SDM with our patients. In our opinion, a well-structured and conducted SDM is now required in *EGFR-ALK* oncogene addicted NSCLC. Also, a well-conducted SDM often does not achieve a really shared decision, because patients often are not able or refuse to fully participate, but at least it leads to a better knowledge of the person that we have ahead and to a better physician’s treatment choice for that single person. For the first time in the history of the treatment of *EGFR* mutant and *ALK* rearranged NSCLC, an improvement in efficacy outcomes is associated with a worsening of toxicity profiles, potentially affecting substantially the quality of life of the patients. For the first time in oncogene addicted NSCLC treatment, physicians and patients together have to face and share the decision to take the road of maximal survival prolongation at the cost of higher toxicity or the road of the preservation of the quality of life, trading off a certain amount of survival time.

## Data Availability

Not applicable.
